# Critical Factors Involved in Primordia Building in *Agaricus bisporus*: A Review

**DOI:** 10.3390/molecules25132984

**Published:** 2020-06-29

**Authors:** Johan J. P. Baars, Karin Scholtmeijer, Anton S. M. Sonnenberg, Arend van Peer

**Affiliations:** Plant Breeding, Wageningen University and Research, PO Box 386, 6700AJ Wageningen, The Netherlands; Karin.Scholtmeijer@wur.nl (K.S.); Anton.Sonnenberg@wur.nl (A.S.M.S.); Arend.vanPeer@wur.nl (A.v.P.)

**Keywords:** *Agaricus bisporus*, primordium building, fruiting regulation, cultivated mushroom, ethylene, 1-octen-3-ol, transcription factor

## Abstract

The button mushroom *Agaricus bisporus* is an economically important crop worldwide. Many aspects of its cultivation are well known, except for the precise biological triggers for its fructification. By and large, for most basidiomycete species, nutrient availability, light and a drop in temperature are critical factors for fructification. *A. bisporus* deviates from this pattern in the sense that it does not require light for fructification. Furthermore its fructification seems to be inhibited by a self-generated factor which needs to be removed by microorganisms in order to initiate fruiting. This review explores what is known about the morphogenesis of fruiting initiation in *A. bisporus*, the microflora, the self-inhibitors for fruiting initiation and transcription factors involved. This information is subsequently contrasted with an overall model of the regulatory system involved in the initiation of the formation of primordia in basidiomycetes. The comparison reveals a number of the blank spots in our understanding of the fruiting process in *A. bisporus*.

## 1. Introduction

*Agaricus bisporus* is one of the most abundantly cultivated mushroom species worldwide [1]. In Europe and North America it is the prime mushroom species. It’s commercially available in various shapes and sizes [2,3]. Fresh *A. bisporus* mushrooms can be found in different colours (white, off-white, brown), sizes (ranging from small mushrooms with 15 mm cap diameter to large mushrooms with a cap diameter of 50 mm and even larger) and levels of maturation (fully closed caps up to fully opened caps). Typical examples are the ‘chestnut mushroom’, the common white, flats, and Portobello. Preserved and processed products of *A. bisporus* include mushroom (blend)burgers, soups, powder, mushrooms in cans or glass, and frozen mushrooms. When cultivating mushrooms, growers are trying to target their mushroom crop towards the desired product types (cap sizes, level of maturation) their customers require. To be able to do so, for growers it is of prime importance to have control over the number of primordia that are formed and the number of primordia that develop into mature mushrooms. These numbers largely determine the size, quality and picking costs of their product.

## 2. Morphogenesis of Primordia

Primordium or fruiting body initiation in *A. bisporus* is generally referred to as pinning. Pinning reflects a multi-stage developmental program, that represents the transition from ‘simple multicellularity’ into compact three-dimensional structures [4,5]. First, vegetative hyphae aggregate into strands [6], (for scanning electron microscope (SEM) images see [7]). Hyphal knots are formed on the strands, and in turn develop into primordia. Formation of the mycelial cords has been described in great detail by Mathew [8], (with drawings) and is further discussed, together with primordium formation by Umar and Van Griensven [9,10,11,12]). As described by Mathew [8], first, hyphae branching from or growing near a leading (larger) hyphae will stop branching away and instead start growing alongside the leading hypha. Next, numerous small non-septate hyphae will emerge from older parts of the leading hyphae. These are referred to as ‘tendril’ hyphae. Tendril hyphae further branch, and start filling up the spaces between larger hyphae in the developing strand, and starting to ensheath the strand. Leading hyphae increase in thickness. Branching of the strands seems to happen by chance when tendril hyphae branch away from the developing strand, or when encountering another leading hypha crossing their path. The mycelial cord is surrounded by fluffy white hyphae that grow vegetative. The hyphae are covered by oxalate crystals, which arise within the hyphae [13]. This biomineralization is observed in many fungi, but its function remains debated (e.g., [14]). The hyphae in the centre of the cord are held together by an extracellular matrix, which aids in creating a three-dimensional pseudoparenchymatous structure (for images see [7]). Within the cord tissues, oxalate crystals are no longer present. Mycelial cord formation precedes the development of hyphal knots, and then primordia, which form at the end of the mycelial cord.

**Figure 1:**
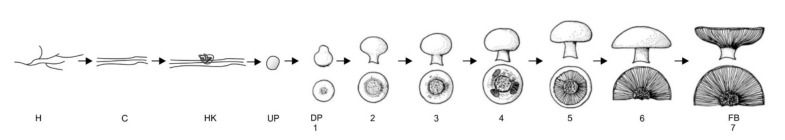
Main stages in the development of a Fruiting body (FB) from hyphae (H). The intermediate stages are cords (C), Hyphal knots (HK), undifferentiated primordia (UP) and from differentiated primordia (DP) to Fruiting bodies (FP) in 7 stages (adapted from Hammond and Nichols [19]). Especially the very early stages are difficult to study in casing soil due to their microscopically small size.

The development of primordia is a gradual process in which the different phases are difficult to discern. Next to this, the number of primordia that develop in the casing soil far exceeds the number of mushrooms produced [15]. Noble et al. [16] found that a commercial strain produced 36,700 primordia and 1300 fruit bodies per square meter of casing soil. Straatsma et al. [17] found the number of primordia to be between 30,000 and 90,000 m^−2^. This high number of primordia is sufficient for all mushrooms that develop in the successive flushes and flushing is the consequence of the depletion of some unknown specific nutrition required by outgrowing primordia [17]. Further development of a primordium into a mushroom is well described by Hammond and Nichols [18,19]. They discriminate seven stages in the outgrowth of the mushrooms. In stage 1 (Pinhead), the cap diameter is less than 5 mm and the velum is not differentiated. At stage 2 (Button), the cap diameter is 20–30 mm, while the velum is visible and intact. At stage 3 (Closed cup), the cap diameter is 30–40 mm, while the velum is stretched but still intact. At stage 4 (Cup), the cap diameter is 30–40 mm, but the velum is starting to tear. At stage 5 (Cup) the cap diameter is 30–50 mm. The velum is torn but the cap is still cup shaped and the gills are clearly visible. At stage 6 (Flat) the cap diameter is 40–60 mm. The surface of the cap is convex while the gill surface flat or slightly concave. At stage 7 (Flat) the mushroom is fully mature. The gill surface is curving upwards exposing the lamellae fully. This classification for maturation of the mushroom is adopted by most scientists. However, a clear classification in different stages is lacking for the development of primordia. In their study on the environmental factors governing fruit body formation in *A. bisporus*, Eastwood et al. [20] discriminated the stages shown in Figure 1. Fluffy mycelial cords had a diameter of about 60 μM. The next stage in their developmental scheme are the fluffy 0.5–1 mm diameter hyphal knots. These were followed by the development of fluffy undifferentiated primordia (1–2 mm diameter, which turned into smooth undifferentiated primordia (2–4 mm diameter). These developed into elongated differentiated primordia (4–10 mm diameter) showing a distinct ‘waist’, separating the developing cap and stipe tissues. This last stage coincides with the pinhead stage according to the classification of Hammond and Nichols [18,19].

## 3. Environmental Factors Influencing Primordia Building

A grower can influence the amount of pins that are produced by his/her choice of casing soil, venting regime and watering regime. By these cultivation measures the grower is influencing in multiple ways the basic mechanisms underlying the process of primordia building. Basically the critical factors involved in formation of primordia by *Agaricus bisporus* are a lowering of both temperature and the level of carbon dioxide in the air of the growing room, as well as the degradation of a volatile self-inhibitor for fruiting. The hypothesis is that this volatile self-inhibitor is either removed by venting the air in the growing room, or by degradation by the microflora present in the casing soil (or a combination of both) [21].

While much is known of the precise control of climate parameters, such as temperature, humidity and carbon dioxide concentrations which are optimal for the growth, far less is known of the mechanisms by which these factors interact to induce mushroom fruiting. Sakamoto [22] provides an overview of the environmental factors that are involved in induction of fruiting and outgrowth of fruiting bodies in basidiomycetes. By and large, for most species, nutrient availability, light and a drop in temperature are critical factors. In many fungi, the development of primordia is triggered by lower nitrogen and carbon concentrations. Next to this, cyclic adenosine monophosphate (cAMP), cerebrosides, light and temperature are involved. However, precise details of the effect of the individual environmental factors on fruiting body induction in basidiomycetes remain to be determined.

*A. bisporus* deviates from the majority of the cultivated basidiomycetes in that it does not require light for fructification. Furthermore, no matter the composition and exhaustion of nutrition in the compost, *A. bisporus* does not shift towards fruiting body initiation or mushroom production directly on the substrate. It needs a special nutrient poor layer, called casing soil. This layer serves as a support for the mushrooms, a water reservoir and a substrate for the development of bacteria (discussed later).

There is a genetic factor, strains differ in their ability to pin [23,24]. Some strains are pinning rather easily, others have more difficulty to pin. However, all *A. bisporus* strains have in common that they do not fruit axenically on an agar based medium.

Eger [25] demonstrated that *A. bisporus* is able to produce primordia and fruiting bodies on a sterilised compost that is covered with active charcoal. Since then, a number of researchers have used activated charcoal to determine conditions that influence fruiting. Both Long & Jacobs [26] and Couvy [27] demonstrated that in sterile casing containing activated charcoal, carbon dioxide control of both vegetative growth and fruiting body initiation in *A. bisporus* closely parallels that observed in unsterilized peat based casing soil. According to Long and Jacobs, it suggests that the adsorbent action of the activated charcoal can serve as a model for the microbial effect in inducing mushroom formation. Noble et al. [16] tested fructification of wild and commercial strains of *A. bisporus* that were cultured in axenic microcosms, using a rye grain substrate covered by a range of organic and inorganic casing materials. In axenic culture, *A. bisporus* (commercial strain A15) produced primordia and mature sporophores on charcoal (wood and activated), anthracite coal, lignite/calcium carbonate and zeolite, but not on bark, coir, peat/calcium carbonate, rockwool, silica or vermiculite. From this Noble et al. [16] concluded that inhibitors prevent initiation of primordia and that the putative inhibitors can be adsorbed by certain casing materials. Noble and Dobrovin-Pennington [28] used this information to develop a peat substitute for use in casing soil, based on fine particle coal tailings (a predominantly rock particle by-product obtained from dewatered suspensions in coal washing plants, with similar clay-like texture to sugar beet lime). Bechara et al. [29] tested activated charcoal as a component of casing soil in their efforts to develop a system for mushroom cultivation based on non-composted substrates [30,31]

### 3.1. Role of Casing Soil Microflora

According to the hypothesis, the microflora in the casing soil removes a volatile self-inhibitor for fruiting, with the bacterial species *Pseudomonas putida* being mentioned as a prime example of a bacterial species able to remove the self-inhibitor [21]. Several authors have shown that a bacterial population develops in the casing soil when it becomes colonized by *Agaricus bisporus* [32,33,34,35]. Scanning electron microscopy showed that several shapes of bacteria attached themselves to the surface of the hyphae; rods, vibrio-like rods, and cocci. Some were attached by filaments to each other and to the mycelial hyphae [33,36]. The bacteria were proposed to be attracted to nutrients in exudates from the fungus. Indeed, *Pseudomonas putida* and *Pseudomonas. tolaasii*, two bacteria that live in close association with mycelia of *A. bisporus*, attached to hyphae of *A. bisporus* and exhibited a chemotactic response towards its exudate. The presence of the sugars in the exudate (glucose, mannose and rhamnose) was of lesser importance; the bacteria responded mostly to the presence of the amino acids glutamine, alanine, leucine, phenylalanine and proline in the exudate [37,38]. In addition, *P. putida* strain UW3 was shown to exhibit strong chemotaxis towards arginine, succinic acid and 1-aminocylopropane-1-carboxylic acid (ACC), a precursor of ethylene production [39]. In addition, it was shown that not only water-soluble compounds in the exudate of *A. bisporus* are consumed by bacteria, but also gaseous compounds that are produced in the mycelia [40].

Reddy and Patrick [41] noticed a steady increase of culturable bacterial populations in a peat-based casing soil with a maximum (100-fold) at the moment of pinning after which the numbers decreased again. Similarly, Cai et al. [34] showed that the development of culturable bacteria in casing soil (composed of paddy soil and rice hulls) increased nearly 100 times in the period between application of casing soil and pin-formation of the first flush. Next to the trend in numbers of culturable bacteria, Cai et al. [33] monitored the microbial community in the casing soil via PLFAs (extractable phospholipid fatty acids). The PLFA profiling as a fingerprint of the casing soil microbial community is regarded to provide a more sensitive measure of change than the more traditional method of culturable bacteria. In the casing soil, PLFAs increased eight times from the time of application of the casing soil to the time of pinning for the first flush, suggesting that at casing the majority of the bacterial community consisted of non-culturable species. After the first flush the bacterial PLFA decreased to a level of about one fourth of the level at the time of picking the first flush. Thus, a change in microbial diversity is associated with mushroom mycelium growth and primordia development. 

Carrasco et al. [42] analyzed the composition of the bacterial population in casing (50:50 blonde peat: black peat) through next-generation sequencing (NGS) of the V3–V4 region of the bacterial 16S rRNA. At the moment of casing, the casing soil contained a variety of bacteria (*Sphingobacterium mizutali*, *Thermomonas fusca*, *Bdellovibrio bacteriovorus*, *Pseudoxanthomonas mexicana*, *Paracoccus aminovorans*, *Desulfosporosinus meridiei*, *Prosthecobacter debontii*, *Brevundimonas diminuta*, *Tetrathiobacter kashmirensis* and *Clostridium intestinale*). Upon colonization of the casing soil by *A. bisporus*, the composition of the bacterial population changed. Species resembling *P. aminovorans*, *D. meridiei*, *B. diminuta*, *T. kashmirensis* and *C. intestinale* lost their abundancy (meaning that they were no longer among the ten most abundant communities in percentage) and were replaced by others. (*Asticcacaulis biprosthecium*, *Methylotenera mobilis*, *Acidovorax delafieldii*, *Sphingopyxis alaskensis*, *Nitrosovibrio tenuis*, *Flavobacterium succinicans*, *Spirochaeta aurantia* and *Phaeospirillum fulvum*). Once the production of mushrooms started, *B. bacteriovorus* and *P. mexicana*, which were already present during casing, remained the dominant bacterial species. In addition, the relative abundance of *Pseudomonas* in the casing increased during the crop cycle, with *Pseudomonas* being the second most abundant genus by the end of the second flush.

Analysis of 16S rDNA present in casing soil at three different mushroom farms showed that no large differences in bacterial populations exist between the casing soil samples from different farms/suppliers. Differences were seen in the bacterial populations on the mycelial strands as compared to the surrounding casing soil. Bacterial species within the genera Flavobacterium and Sphingobacterium were found both on the mycelial strands and in the surrounding casing soil, the genus *Pedobacter* being present mostly on the mycelial strands. Within the phylum Proteobacteria, the abundance of the alpha-proteobacteria and the beta-proteobacteria was higher in the casing soil than on the mycelial strands. Within the beta-proteobacteria, the differences in location were most prominent for the *Burkholderiales*, especially with respect to the *Comamonadaceae*. The gamma-proteobacteria were more prominent on the mycelial strands, especially the *Pseudomonadales*.

In casing soils of South African reed-sedge peat, and the industrial waste materials coir, wattle bark, bagasse and filter cake, dominant bacterial species from pinning onwards were shown to be close relatives of *Flavobacterium* spp. and *Pseudomonas* spp. Next to these, close relatives of alpha-proteobacteria, beta-proteobacteria, gamma-proteobacteria, delta-proteobacteria and uncultured species were abundant [43]. The microbial diversity in the samples was in this case studied via sequencing of dominant protein bands in a DGGE (denaturing gradient gel electrophoresis) fingerprint pattern followed by a basic local alignment search tool (BLAST) search for each sequence. 

Thus the amount of bacteria increases upon casing colonization with *A. bisporus*, and although bacterial populations may differ in composition between different types casing layers and even between the mycelial strands or the surrounding casing, the general trend is the presence of *Pseudomonas* species. 

### 3.2. Production of Volatile Organic Compounds 

**Figure 2:**
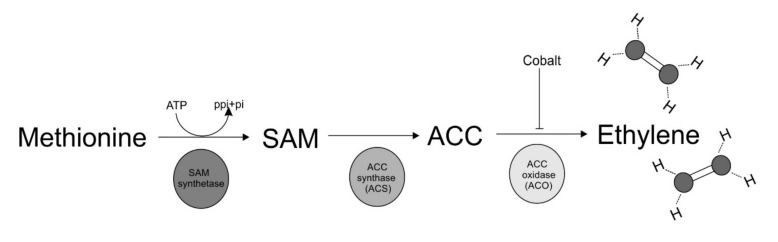
Global overview of the pathway for production of ethylene by *A. bisporus*. SAM = *S*-adenosyl-l-methinine; ACC = 1-aminocyclopropane-1-carboxylic acid. Adapted from Zhang et al. [57].

The compound that inhibits fruiting by *A. bisporus* is believed to be a volatile organic compound produced by the organism itself [21]. Next to carbon dioxide, *A. bisporus* has been reported by various authors to produce acetic acid, acetaldehyde, acetone, amylalcohol, benzaldehyde, benzyl alcohol, n-butane, two unspecified isomers of butene, n-butanol, 3-methylbutanol, butyric acid, carbonyl sulphide, carbon disulphide, ethane, ethanol, ethylene, ethyl acetate, ethyl methyl ketone, n-hexanal, isobutyric acid, isovaleric acid, isobutanol, iso-butane, isoamylalcohol, methyl chloride, n-pentane, propane, propene, a number of C_8_-compounds (octan-3-one, octan-3-ol, oct-1-en-3-ol, oct-1-en-3-ene, oct-2-en-1-ol), 2,3-decadienal, 2,3-nonadienal, an unidentified sequiterpenoid, tetrachloro-1,3-dimethoxybenzene and valeric acid [44,45,46,47,48], either as vegetatively growing mycelium on a number of substrates, basidiospores or as fruiting bodies. Tschierpe and Sinden [49] demonstrated that a number of these compounds were a result of anaerobic metabolism (acetone, ethanol, acetaldehyde, and ethyl acetate). Turner et al. [47] demonstrated that ethylene showed a pattern of production that could be correlated with developmental phases of the crop, high levels being produced whenever fruit bodies were rapidly enlarging (thus after primordium formation). Production of ethylene occurred within the colonized compost; no ethylene was evolved by the fruit body itself as evidenced by the fact that after picking the mushrooms, there was still production of ethylene comparable to the rate seen in the controls in which mushrooms were not picked. Wood & Hammond [50] studied the production of ethylene in cultures of *A. bisporus* grown in jars on sterilized compost, which was covered with a layer of sterile, activated charcoal granules. They were able to demonstrate that the ethylene was indeed produced by the *A. bisporus* mycelium and not by accompanying micro-organisms. A rise in ethylene production always coincided with the development of one or more fruiting bodies in a particular jar. Ward et al. [51] demonstrated that ethylene was produced by mycelium in compost. No ethylene was produced by mycelium in the casing soil or by bacteria. Based on the fact that methionine stimulated the production of ethylene in *A. bisporus* [51], Chen et al. [35] postulated a similar pathway for the production of ethylene as present in plants. The ethylene biosynthesis pathway in plants consists of two steps. In a first reaction, S-adenosyl-L-methionine (SAM) is converted into 1-aminocyclopropane-1-carboxylic acid (ACC) by ACC-synthase (ACS). In a second reaction, ACC is converted into ethylene by ACC-oxidase (ACO) (Figure 2, [52]). ACC was detectable in the hyphae of *A. bisporus* and when supplemented with 5 mM methionine or cobalt chloride (an inhibitor of ACC-oxidase), the ACC concentrations within the hyphae were significantly increased. This indicates indeed a similar route of ethylene production as compared to plants.

Pfeil and Mumma [53] sampled volatiles from *A. bisporus* from the air in growing rooms of a mushroom farm during the course of four cultivation cycles (during the period from spawning till pinning). As their primary goal was to find which volatiles attracted for female Phorid flies (*Megaselia halterata*), they focused on 3-octanone and 1-octen-3-ol and did not report on the presence of ethylene. No detectable amounts of 3-octanone and 1-octen-3-ol were emitted during the first 8 to 9 days after spawning. After this first period the results varied a bit between the four crops that were sampled. At 12 to 13 days from spawning, small amounts of 3-octanone were emitted and sometimes a small amount of 1-octen-3-ol was detected. Large amounts of 3-octanone were emitted when casing soil was applied with compost mixed into it (a practice called Compost Added Casing (CAC-ing); aimed at reducing the time needed to have the casing soil colonized with *A. bisporus* mycelium to about 5–7 days). In addition, low levels of 1-octen-3-ol were found. The level of 3-octanone dropped rapidly 5 to 6 days after CAC. No 1-octen-3-ol was detected at this time. The rooms were vented 6 days after casing and CAC-ing. The authors noted that the physical breakup of spawn-run compost while performing CAC-ing releases both 3-octanone and 1-octen-3-ol. They assume that the rupturing of fungal cells releases the enzymes that split the supposed precursor, linoleic acid, to form the volatiles. Combet et al. [54] propose that linoleic acid is oxidized to form a 10-hydroperoxide intermediate, which is then cleaved to form 1-octen-3-ol and 10-oxo-trans-8-decenoic acid, (10-ODA). Pfeil and Mumma [53] remarked that the peat moss layer spread onto spawned compost at CAC moderates the amounts of 3-octanone and 1-octen-3-ol released and that there appears to be a selective absorption of 1-octen-3-ol by the peat moss.

Noble et al. [45] studied the production of volatile organic compounds during the formation of primordia by *A. bisporus* in microcosm cultures containing 30 g of sterile rye grain spawn which was covered either with axenic activated charcoal or non-axenic peat/calcium carbonate based casing. More than 30 volatile compounds were identified, but volatiles that were present only in the microcosms inoculated with *A. bisporus* were exclusively 8-carbon compounds, mainly 1-bromo-octane, 1,6-octadien-3-ol, octane, 1-octanol, 3-octanone and 1-octen-3-ol. 

Researchers have struggled for decades to find out which of these volatile organic compounds are relevant for the induction of fruiting by *A. bisporus*. Most did not appear to be relevant, except ethylene, ethanol, and the C_8_-compounds.

#### 3.2.1. The Effect of Ethylene on Pinning 

Ethylene is believed to be a potential candidate for the role of self-inhibitor of fruiting in *A. bisporus*. Physiological effects of ethylene on *A. bisporus* were difficult to demonstrate [51]. The growth rate of mycelium on compost extract agar showed no significant differences between cultures treated with 10, 100 or 1000 ppm of ethylene and the controls in which ethylene was depleted, nor was there any visible difference in the morphology of the mycelium grown in these atmospheres. Ethylene administered at 10 ppm to the air of cultures in compost, covered with casing also did not have any influence on pinning and subsequent maturation of the mushrooms. Next to this no interactions were found between ethylene and carbon dioxide. Visscher [55] tested the effect of ethylene on fructification in *A. bisporus* in glass dishes containing a layer of compost, covered by a casing soil by adding either 0, 1 or 10 ppm ethylene to the air stream with which cultures were vented. Outgoing air from the boxes was led away by a duct. As he was looking at a postulated carbon dioxide/ethylene antagonism, he performed his experiments either in the presence of ambient levels of carbon dioxide or increased amounts. Ethylene in the air mixture at a level of 1 ppm stimulated fructification, by increasing the number of primordia formed in *A. bisporus*, in combination with carbon dioxide at ambient air concentration [54]. The response to ethylene at a level of 10 ppm depended on the level of carbon dioxide. At higher levels of carbon dioxide there were slightly more pinheads with 10 ppm ethylene after 13 days, whereas with at lower levels of carbon dioxide numbers of pinheads were equal or somewhat less. Based on these results he postulated that ethylene and carbon dioxide should be in the right balance to promote pin head formation in *A. bisporus*.

Kurtzman [56] describes the effects of cobalt (an inhibitor of ethylene production), with or without the addition of ethylene in the atmosphere, on fruiting of *A. bisporus*. In his experiments he used colonized compost which was covered with casing soil to which various amounts of cobalt chloride was added. The trays used for ethylene experiments were placed in acrylic chambers that were aerated with humidified air containing approximately 0.02% ethylene (200 ppm). All concentrations of cobalt caused a decrease in yield and a delay in fruiting, thus lowering the ethylene concentration reduced primordia formation. The effect of ethylene was also tested at a concentration of 0.8 mM of cobalt chloride in the casing soil, which also caused a decrease in yield and delay in production. The addition of ethylene resulted in the first mushroom production occurring 12 days earlier than without ethylene (which is about the same time of production on the casing soil without 0.8 mM cobalt chloride). This indicated that ethylene in the air may stimulate primordium development.

In contrast, an inhibitory effect of ethylene on pinning was demonstrated by Zhang et al. [57]. They suppressed endogenous production of ethylene by downregulation of the ACO-gene via RNA interference The mycelial growth rate of six ACO-downregulated transformants on sterilised compost was significantly higher than that of the wild-type strain. In contrast to the wild-type, a tested transformant formed primordia normally in sterilized casing soil. In addition, on a non-sterilised casing soil, the number of primordia formed by the transformant was 79.4% higher than that of the wild-type strain This indicates that lowering the ethylene production induces/stimulates primordia formation. 

These results seem to be in contrast with those of Ward et al. [51], Kurtzman [56] and Visscher [55] where primordium formation was unaffected or stimulated by adding exogeneous ethylene. It should be noted that Cobalt chloride also has an effect on bacterial growth, and thus the microbiome of the compost might have been altered. Similarly, down regulation of a metabolic enzyme can have several additional effects. Thus, lowering the turnover of ACC to ethylene has a stimulatory effect on primordium formation. These results can’t be compared without additional studies.

#### 3.2.2. The Effect of C_8_-Compounds on Pinning

Pinning in *A. bisporus* appears in most cases to be inhibited by volatile organic compounds. The influence of volatile organic compounds (VOC) on the formation of primordia by *A. bisporus* was examined by Noble et al. [45] in microcosm cultures (30 g of sterile rye grain spawn covered with axenic activated charcoal or non-axenic peat/calcium carbonate based casing). Addition of 8-carbon compounds to the venting air on the colonization of casing soil and the formation of primordia were analysed. The air flow to the microcosms contained ethanol, 2-ethyl-1-hexanol, octane, 3-octanone, 1-octen-3-ol or cis-3-octen-1-ol. Cultures aerated without VOC were used as controls. The presence of ethanol or C_8_-compounds reduced mycelial growth in the casing. The presence of ethanol or 1-octen-3-ol almost completely inhibited primordium formation. Primordium formation was less affected but still suppressed by 2-ethyl-1-hexanol and cis-3-octen-1-ol. Octane suppressed the numbers of primordia but not of number of outgrowing fruiting bodies and 3-octanone had no significant effect on either number of primordia or mushrooms. After the removal of the volatiles from the air stream into the jars, primordium formation occurred within 7 days and at least one mushroom per microcosm was produced in all treatments. This seems to indicate that ethanol, 1-octen-3-ol, 2-ethyl-1-hexanol, cis-3-octen-1-ol and to a minor extent octane are able to negatively influence pinning in *A. bisporus*. 

### 3.3. Interactions between the Production of Volatile Compounds and Bacteria

The importance of bacteria for pinning of *A. bisporus* has been demonstrated already since the 1960′s [25,58,59,60]. The “Halb schalen test”, a test in which a Petri dish is half filled with compost and half filled with casing soil showed that a sterile casing soil does not allow the development of mushrooms, while on the non-sterilized casing soil mushrooms developed [25]. When a suspension of casing soil was filtered through a paper filter, and the filtrate was added to the sterile casing soil, *A. bisporus* started to produce pins. If bacteria were filtered out of the suspension, no pinning was obtained on a sterile casing soil. From this Eger concluded that the bacteria, and not some soluble compound, promoted pinning. A similar result was obtained using Petri dishes with 4 quadrants. Two opposite quadrants were filled with colonized compost, while the two adjacent quadrants contained sterile casing soil. Primordia were only formed in the quadrant filled with sterile casing soil which was sprinkled with a suspension made from non-sterilized casing soil [61]. This suggested that bacteria need to make physical contact with the mycelial strands in order to allow pinning.

A number of authors have tried to identify which bacterial species were involved in promoting pinning in *A. bisporus*. Originally these studies focused on bacterial species that could be cultured. With the advent of molecular techniques researchers have also tried to study the bacterial species that cannot be cultured. Hume and Hayes [62] demonstrated in Petri dishes containing malt extract agar that *Pseudomonas putida* influenced mushroom initiation in *A. bisporus*. Likewise, Eger [63] demonstrated that *P. putida* strains were able to induce pinning in halb schalen tests. However, in comparison with the effect of a bacterial population isolated from mushroom beds, the effect of the *Pseudomonas* strains was weak, indicating that *P. putida* is not the only factor influencing pinning. Next to *Pseudomonas* strains such as *P. putida* [64], also strains of *Alcaligenes* sp. [65], *Bacillus* sp. [60,66], *Rhodopseudomonas palustris* [67], *Azotobacter vinelandii*, *Rhizobium* sp., the green algae *Scenedesmus quadricauda* and the yeast *Lipomyces starkeyi* [66], were reported to stimulate formation of primordia.

It was proposed earlier [63] that bacteria play a role in pinning via degradation of volatile compounds that inhibit primordia formation. The influence of *Pseudomonas* strains and their effect on the concentration of volatile organic compounds on the formation of primordia by *A. bisporus* was examined by Noble et al. [45] in glass microcosms containing a 10 mm layer (30 g) of sterile rye grain spawn covered either with an axenic or non-axenic casing. The *A. bisporus* strains A15 (Sylvan) and 5776 (Le Lion, Varrains, France) were selected on the basis of known differences in primordium formation in culture. In this system, isolates of *P. putida* (5 isolates), *P. veronii* (2 isolates), *P. poae* and an unnamed *Pseudomonas* species (2 isolates) were tested for their ability to induce pinning. Bacterial suspensions were inoculated onto the axenic casing in the *A. bisporus* microcosm cultures. In the control (axenic casing without one of the *Pseudomonas* isolates), no primordia were formed. The two *P. veronii* isolates had little or no stimulatory effect on primordium formation. The *P. poae* isolate and one strain of the unnamed *Pseudomonas* species performed best. The 5 isolates of *P. putida* showed an intermediate performance. The influence of volatile organic compounds in combination with the presence of bacteria on primordium formation in *A. bisporus* was also examined in the microcosm cultures. A single addition of the 8-carbon compounds 1-octen-3-ol, 2-ethyl-1-hexanol, 3-octanone, trans-3-octene, 1-octanol or octane to the atmosphere in the cultures, did not affect mycelial growth in the casing. The numbers of primordia that formed either on axenic activated charcoal or non-axenic peat based casing in the presence of the various C_8_-compounds did not vary from the control, with the exception of 2-ethyl-1-hexanol. The presence of 2-ethyl-1-hexanol in the microcosms suppressed primordium formation. However, 2-ethyl-1-hexanol turned out to be a volatile that is produced by the uninoculated rye grain spawn and therefore not by the *A. bisporus* mycelium. Final total bacterial numbers in the peat based casing were higher in the presence of 1-octen-3-ol and 2-ethyl-1-hexanol as compared to the control microcosms and in the presence of the other C_8_-compounds. The *Pseudomonad* population was increased by the presence of 2-ethyl-1-hexanol in the microcosms. On the axenic charcoal casing, more primordia were formed than on the non-axenic peat based casing. Continuous addition of the 8-carbon compounds 1-octen-3-ol, 3-octanone, trans-3-octene, 1-octanol and octane to the air with which microcosms were vented, was tested using sterilized compost as a substrate and either axenic or non-axenic peat/calcium carbonate based casing. As mentioned above, the presence of ethanol or 1-octen-3-ol almost completely inhibited primordium formation, which also was suppressed by cis-3-octen-1-ol. Octane suppressed the numbers of primordia but not of sporophores and 3-octanone had no significant effect on either number of primordia or sporophores. After the removal of the volatiles from the air stream into the jars, primordium formation occurred within 7 days and at least one sporophore per jar was produced in all treatments. The *Pseudomonad* population of the casing in the control treatment remained stable during the *A. bisporus* culture period although the total numbers of bacteria declined. 1-Octen-3-ol increased the total bacterial population of the casing during the culture period and all the volatiles except 3-octanone increased the population of *Pseudomonads* in the casing. Noble et al. [45] conclude that a reduction in carbon dioxide concentration is not the sole requirement for primordium formation of *A. bisporus*. The stimulatory effects of the casing and its microbial population and air exchange on primordium formation of *A. bisporus* are due at least partly to the removal of inhibitory C_8_-compounds produced by the mycelium and its substrate. They advise experimentation aimed at separating the independent effects of inhibitory volatiles and carbon dioxide on primordium formation.

Different mushroom strains respond differently to the same bacterial isolate [41]. A number of predominant bacterial types observed on dilution plates were tested for their ability to induce primordia formation in four *A. bisporus* strains using Halb schalen tests as described by Eger [68]. Primordia were visible 16–22 days after casing when these bacteria were present. In addition, the presence of bacterial suspensions in the casing layer was inhibitory to the growth of mushroom mycelium but induced strand development and stimulated formation of primordia.

Chen et al. [35] demonstrated how *Pseudomonads* influence the level of ethylene produced by *A. bisporus* mycelium. *Pseudomonads*, including *P. putida*, are capable of producing the enzyme 1-aminocyclopropane-1-carboxylic acid (ACC) deaminase (AcdS) [69,70]. This enzyme which catalyzes the cleavage of ACC to ammonia and 2-oxobutyrate, thus decreasing ethylene levels. Two strains of *P. putida* were used to test the effects of AcdS activity on hyphal growth and primordium formation of *A. bisporus*. One strain containing AcdS (AcdS^+^), while the other is an AcdS-deficient (AdS^−^) mutant (unable to convert ACC and therefore not suppressing the production of ethylene). In co-cultures of *A. bisporus* with either *P. putida* AcdS^+^ or AcdS^−^, it was seen that lowering of the ethylene production via using the AcdS^+^ strain resulted in a stimulatory effect on primordia formation. The number of pins of *A. bisporus* with *P. putida* AcdS^+^ in sterilized casing soil was even higher than that of un-sterilized casing soil in the first flush, but not different in the second. Indicating the strong effect of the *P. putida* AcdS^+^ strain in lowering ethylene production and thus inducing pinning. Next to this, Chen et al. [35] were also able to demonstrate that Pseudomonads are not the only bacterial species in the casing soil that carry the AcdS gene. In their experiments they showed that from casing application to hyphae half-penetration of casing layer, the counts of total culturable bacteria and (AcdS) producing bacteria in the casing soil increased to 30 and 60 times. The percentage of AcdS-producing bacteria among the total culturable bacteria reached 20% at the stage of hyphae half-penetration of casing layer, which was considerably higher than the 3–5% in other stages. Based on the fact that the dominant AcdS-producing bacteria within the casing, in this case clay loam sub soil, were not *P. putida*, led them to conclude that the dominant species of bacteria in casing soil are probably influenced by the soil type. The results obtained by Chen et al. [35] seem to contrast with those of Ward et al. [51]. The bacteria with which Chen et al. [35] worked, were added to the casing soil in order to lower the production of ethylene. Ward et al. [51] demonstrated that the production rate of ethylene in compost and casing soil is about equal when no fruiting bodies are being produced. However, when producing fruiting bodies, the production of ethylene in the compost is about 10 times higher than in the casing soil. Turner et al. [47] measured ethylene production in casing soil and compost at various times during the fructification period of *A. bisporus* and found a production rate of 0.07–0.08 nL/g fresh weight of casing/hr when the casing soil was being colonised by the mycelium, with a decrease when pinning was initiated. The question remains as to whether the mycelium in the casing has a lower production of ethylene or whether the bacteria in the casing soil are very effective in removing the precursor for ethylene production from the mycelium. Upon maturation of the fruiting bodies, ethylene production rises steeply and this ethylene is likely to be produced by the mycelium in the compost. The results of Chen et al. [35] also appear to contrast with those of Kurtzman [56] and Visscher [55], who both notice a stimulatory effect of ethylene on pinning, instead of a suppression of pinning.

## 4. An Attempt at Building a Model for the Control of Primordia Building by *A. bisporus*

Eastwood et al. [20] studied the process of pinning using transcriptomics. For this they sampled casing soil and mushrooms both from mushroom compost tray experiments, and from bell jar experiments (as described by Noble et al. [16]). They took samples from very early on in the process of primordia building and took very good care in establishing at what precise moment during the process of primordia building samples were taken. The developmental stages they sampled are shown in Figure 1. In mushroom compost tray experiments, colonised casing was collected, starting at the time of airing (defined as t = 0) until stage 2 fruiting bodies (button mushroom without a stretched veil, see Hammond and Nichols [19]) had developed. In addition, samples were collected of primordia and mushrooms of different developmental stages from control mushroom trays when they were first observed. The bell jar experiments allowed manipulation of the concentration of both carbon dioxide and 1-octen-3-ol separately. In addition, control experiments were conducted to separate the effects of temperature and carbon dioxide. For this, one of the growing rooms was ventilated, but temperature held at 25 °C, while a second growing room was not ventilated (carbon dioxide remained above 3000 ppm), but temperature was reduced to 18 °C. In the bell jar experiments, 350 ppm 1-octen-3-ol was added to the air supply for 8 days, after which a standard air supply was applied. In a separate bell jar system a stream of carbon dioxide gas was added to the air supply to maintain the level of carbon dioxide at approximately 5000 ppm in the exit gas flow. Changes in morphology were monitored on an hourly basis using time lapse photography and stereo microscope positioned directly above the developing fruiting bodies and to the side, in line with the casing layer. In addition, a photographic record was made of the trays in the cultivation rooms where either temperature or headspace gases were not reduced, and in the bell jar experiments where either carbon dioxide or 1-octen-3-ol levels were controlled separately. Morphological changes under standard cultivation conditions were first observed after approximately 32 h following the reduction in temperature and increased ventilation in the growth room, where approximately 60 μM diameter fluffy mycelial cords formed in the casing layer. These cords thickened to form fluffy 0.5–1 mm diameter hyphal knots which developed into fluffy undifferentiated primordia (1–2 mm diameter) by approximately 95 h after venting. After approximately 120 h smooth undifferentiated primordia (2–3 mm diameter) were observed, some of which developed into elongated differentiated primordial structures (3–10 mm diameter) at approximately 133 h. Differentiating primordia with a distinct ‘waist’, i.e., a region of reduced diameter approximately halfway down separating the developing cap and stipe tissues were observed by 200 h after venting which lead to the formation of mature fruiting bodies. Remarkably, the smooth undifferentiated primordia formed on the surface of the casing did not develop further, while the primordia formed below the surface or in cracks in the casing matured into fruiting bodies (a phenomenon also noticed by Straatsma et al. [17]). When the temperature was maintained at 25 °C and the room ventilated to reduce carbon dioxide levels to below 1000 ppm, the developmental changes progressed similarly to the standard condition treatments until the point at which smooth undifferentiated primordia were formed, after which no further development was observed. When the temperature was reduced to 18 °C fruiting continued similar to the control. When the room was not ventilated and temperature reduced to 18 °C, there was some evidence of fluffy cords and early knots in places, but overall no morphological change was observed until ventilation to reduce carbon dioxide levels to below 1000 ppm was applied. Applying 1-octen-3-ol to the airstream entering the flask during phase stimulating conditions resulted in no morphological change, once removed however, fruiting occurred normally. Maintaining carbon dioxide levels in the bell jar experiments at approximately 5000 ppm allowed the developmental changes to occur, but at a reduced rate (two mushrooms formed per flask compared with six under standard conditions). Results of the micro-array analysis showed a total of 45 genes that were associated with early events in fructification, of which 35 were up-regulated during the time course studied and 10 down-regulated. Thirty-one genes were significantly differentially expressed within 23 h of the onset of venting, nine of which showed increased transcription at each sampling point. Comparison of the profiles of the 35 genes identified three clusters of genes with similar transcriptional responses. Cluster 1 genes were associated with later developmental stages, while cluster 2 and 3 were early response genes, mostly differentially regulated within 23 h after venting. Gene cluster 1 describes genes that are putatively involved in processes that occur after smooth undifferentiated primordia have been formed. This is because differential expression was observed only when temperature was reduced and not when high temperature was maintained. Furthermore, the greatest increases in expression of cluster 1 genes occurred after 72 h (3 days) after venting. Next to this, with the exception of the putative major facilitator family transporter, the genes in this cluster were not differentially regulated in bell jar flask cultures where carbon dioxide levels were maintained yet mushrooms still formed, albeit at a reduced rate. Gene cluster 2 comprising of 3 upregulated genes and 1 down-regulated gene which are differentially regulated when fluffy mycelia cords, hyphal knots, and fluffy and smooth undifferentiated primordia are formed. Cluster 2 genes were not differentially expressed in unventilated growth rooms (high carbon dioxide and volatile levels) where development did not proceed beyond vegetative mycelia. Different genes in cluster 2 respond differently to carbon dioxide and 1-octen-3-ol levels. Metallothionein and isoprenylcysteine carboxyl methyl transferase genes were up-regulated in flasks exposed to high carbon dioxide, but not under high 1-octen-3-ol, suggesting the presence of the volatile suppresses their expression. Isocitrate lyase was down-regulated under high 1-octen-3-ol and not under high carbon dioxide, which suggests a response to carbon dioxide levels. The genes in cluster 3 generally showed differential expression under all conditions, regardless whether morphogenetic change has occurred or not, and the expression of all but 3 was unchanged in flasks where the volatiles were removed. The cluster harbours 23 up- and 3 down regulated genes, 23 of which are differentially regulated within the first 23 h post-airing, and 13 showed no increased transcription between 72 (3 days) and 168 h (7 days). The only exception in this cluster was a group of 5 up and 2 down-regulated genes for which a change in expression was not detected in the unventilated growth house. The genes had various functions, including nutritional and metabolic genes, a transporter, structural genes, oxidoreductases and 11 predicted proteins, one of which was not annotated on the *A. bisporus* genome sequence. A set of three genes did not match the profiles of the three clusters above. Two of these genes were not differentially regulated in growth room experiments with maintained high temperature or carbon dioxide levels, but were up-regulated in cultures where either carbon dioxide or 1-octen-3-ol levels were high. In contrast, the glutathione transferase gene was up-regulated in all conditions except in flasks with high carbon dioxide and where 1-octen-3-ol was removed. Using transcriptomics, Krizsan et al. [5] studied gene expression in six other basidiomycete species upon transition from vegetative mycelium to the primordium stage and found Gene Ontology terms related to fungal cell wall functions, oxidoreductase activity, and carbohydrate metabolism to be enriched in genes expressed at fruiting body initiation. Other commonly enriched terms covered functions such as DNA replication, transmembrane sugar transport, and ribosome, membrane, and lipid biosynthesis (while many others were specific to single species). The results obtained by Eastwood et al. [20] are in good agreement with the results of Krizsan et al. [5].

**Figure 3:**
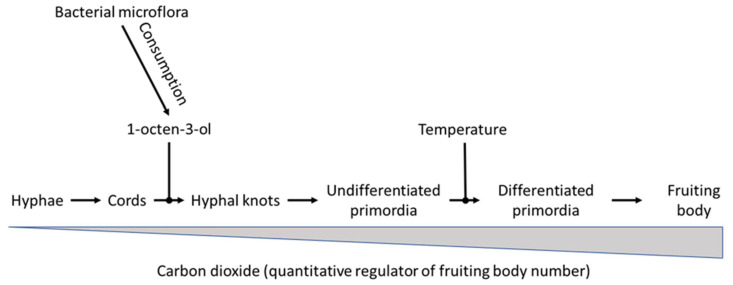
Model proposed by Eastwood et al. [20] of factors influencing the developmental process of fruiting in *A. bisporus*. The volatile 1-octen-3-ol acts as an early repressor of development, while temperature inhibits the transition from smooth to elongated differentiated primordia. The higher the temperature, the less primorida transition from smoot to elongated differentiated primordia. Carbon dioxide is a quantitative regulator of fruiting body number. The higher the level of carbon dioxide in the growing room, the lower the number of fruiting bodies that develop.

Based on the results of the microarray study, Eastwood et al. [20] postulated a model (Figure 3) for the regulation of *A. bisporus* fruiting body production by carbon dioxide, 1-octen-3-ol and temperature. According to that model, 1-octen-3-ol and temperature reduction are the master control switches which are absolute requirements for the complete morphogenesis to fruiting body formation. The volatile 1-octen-3-ol would act early on in the developmental program from undifferentiated hyphae into mature mushrooms, controlling the transition of mycelial cords into hyphal knots, temperature would act later on allowing undifferentiated primordia to further develop into differentiated primordia. Carbon dioxide is seen as a quantitative regulator as it affects the number of fruiting bodies formed by *A. bisporus*, but does not act as an essential regulatory switch. The authors suggest that such a model fits into the conditions that *A. bisporus* might encounter in its natural leaf litter and forest soil habitat, in which carbon dioxide and fungus-derived 8-carbon volatile levels accumulate. Sensing changes in the gaseous environment are postulated to provide a mechanism by which hyphae determine proximity to the surface, and/or emerging fruiting bodies detect points in the soil which allow volatiles to dissipate. In that line of reasoning, the temperature drop that regulates the change from undifferentiated to differentiated primordia is seen as an adaptation by the fungus to fruiting in autumn. According to Eastwood et al. [20], cluster 1 and 2 genes could represent a response to the morphogenetic changes occurring during the reproductive phase change, either by being directly involved in regulating morphological change or by responding to an underlying alteration in cell physiology. Cluster 3 genes, however, are differentially regulated in all treatments and may form part of a genetic response which conditions the *A. bisporus* hyphae for fruiting. The genes in de various clusters code for a range of functions including cell-to-cell interactions, signal transduction, and nutrition. The large proportion of nitrogen metabolism genes indicates a coordinated response in preparation for a surge in demand for new resources by the developing fruiting body. Promoter analysis showed putative conserved sequence motifs common to genes in each cluster, to early response genes (clusters 2 and 3) and to down-regulated genes, suggesting coordinated regulation of genes in response to induction of fruiting by venting. Linking these conserved sequence motifs to transcription factors would allow to make a start to unravel the core regulatory program underlying fruiting in this basidiomycete.

## 5. Transcription Factors Involved in Initiation of Primordia Formation

Homologs of the *Schizophyllum commune* transcription factors involved in fruiting body development [71,72,73] have been identified in *A. bisporus* [73] and are believed to be part of a core regulatory program for mushroom development in the Basidiomycota (with species specific adaptations as suggested by Plaza et al. [74], Gupta et al. [75], Krizsán et al. [5], and Wang et al. [76]). Inactivation of *wc-1* and/or *wc-2* results in a *S. commune* strain unable to sense blue light, which was not able to produce aggregates, primordia, and fruiting bodies [77]. *S. commune* strains in which the homeodomain gene *hom2* or the zinc finger transcription factor gene *fst3* have been inactivated are not able to produce aggregates [71,72]. In contrast, inactivation of the gene encoding the Cys2His2 zinc finger protein *c2h2* results in a strain that does form aggregates but primordia and fruiting bodies are not formed [71]. Strains in which genes are inactivated that encode the zinc finger protein *Fst3*, the GATA type zinc finger protein *Gat1*, or the homeodomain protein *Hom1* form smaller fruiting bodies but in higher numbers [71]. Also in *Pleurotus ostreatus* it was shown that silencing *Pofst3* resulted in higher numbers of primordia, but smaller fruiting bodies [78]. Based on their results, Ohm et al. [71] proposed a model for the relation between the transcription factors and the morphogenesis of mushroom formation in *S. commune*. Differentiation in asymmetrical colonies of *S. commune* proceeds with the development of mushrooms. Transcription factors *bri1* and *hom2* are associated with the differentiation into asymmetrical colonies. These asymmetrical colonies can produce aggregates in which *fst4* is involved. Aggregates develop into primordia where *c2h2* is involved in primordia formation. On the other hand *fst3* is involved in inhibiting the development of primordia. Transcription factors *hom1* and gat1 are involved in the subsequent development into mushrooms.

Pelkmans et al. [79] studied the expression of transcription factors at various stages of fruiting (mycelium in casing soil, 1–2-mm hyphal knots, buttons at 3–5 mm diameter, buttons showing differentiation within the cap and stipe tissue) in a commercial crop of *A. bisporus* strain A15 (unfortunately their terminology and classification of the various developmental stages differs from that of Eastwood et al. [20], which makes comparison more difficult). Expression of the *A. bisporus* orthologues of the blue light sensor gene *wc-1* and the transcription factor genes *wc-2*, *hom2*, *fst3*, *c2h2*, *fst3*, *gat1*, and *hom1* of *S. commune* [73] was analysed at the different stages of development and compared with mycelium in the casing layer. Transcript levels of *wc-2* and *c2h2* increased more than 2-fold in 1–2 mm hyphal knots compared to casing mycelium, while *hom1* levels decreased more than 2-fold. Genes *hom2* and *fst3* were approximately 2-fold upregulated when 1–2 mm hyphal knots had developed into buttons at 3–5 mm diameter. Gene *c2h2* showed high expression at different stages of fruiting body development. Expression levels of 3-fold and more were observed in 1–2 mm hyphal knots, caps of buttons at 3–5 mm diameter, gill tissue of larger buttons, and veil tissue of young fruiting bodies compared to casing mycelium. Expression of *c2h2* was reduced 20-fold and more when compared to casing mycelium in stipe and cap skin and in inner cap tissue of young fruiting bodies. Increased (2-fold or more) levels of *fst3* were only observed in stipes of buttons at 3–5 mm diameter and in stipe skin and tissue of larger buttons. Genes *hom1*, and in particular *gat1* were in general downregulated when compared to casing mycelium. *A. bisporus* gene *c2h2* was introduced into *A. bisporus* strain A15, resulting in a number of transformants, 2 of which showed a 30- and a 2.5-fold increase in *c2h2* expression. Mushroom production of these two transformants was studied in trays using commercially prepared compost and casing soil, with commercial strain A15 as a control. Morphology, cap expansion rate, and total number and biomass of mushrooms were not affected by overexpression of *c2h2*. However, yield per day of the *c2h2* overexpression strains peaked 1 day earlier. The fact that expansion rate of mushrooms was similar between the transformants and A15 implies that accelerated mushroom formation is caused at the level of outgrowth of initials. Even though there is an effect of overexpression of *c2h2* on the time course of production, there was not a very large effect on numbers of primordia. Wu et al. [80] found that orthologs of some of the transcription factors with regulatory function in fruiting in the two model species *S. commune* and *Coprinopsis. cinerea*, did not display expression patterns correlating with their orthologs in industrially cultivated mushrooms (among which *Flammulina. velutipes*, *P. ostreatus*, *A. bisporus* and *Lentinula. edodes*). They propose that fungi in the phylum Basidiomycota may have diverse regulatory mechanisms in basidioma development. They have identified a transcription factor *pdd1* which has a homolog in *A. bisporus*. Transcription of *pdd1* was strongly induced during the development of basidiomata. When *pdd1* was overexpressed, the biomass of *F. velutipes* fruiting bodies produced was increased, while the cultivation time was shortened. Fruiting body development was dramatically decreased by *pdd1* knockdown. In *F. velutipes*, *pdd1* likely controls basidioma development through regulating the expression of genes encoding jacalin-related lectins. Next to this, *pdd1* might exert its influence on basidioma development through regulating a pheromone pathway. It is currently unknown whether *A. bisporus* has an ortholog of *pdd1*.

Even though there are examples of genes being upregulated or downregulated in *A. bisporus* [39,79,81,82,83], genetic transformation of *A. bisporus* is not easily achieved. This may hamper the functional analysis of transcription factors that potentially are involved in the developmental processes of fructification.

## 6. Building an Overview of Pinning and the Regulation of Involved Processes in *A. bisporus*

**Figure 4:**
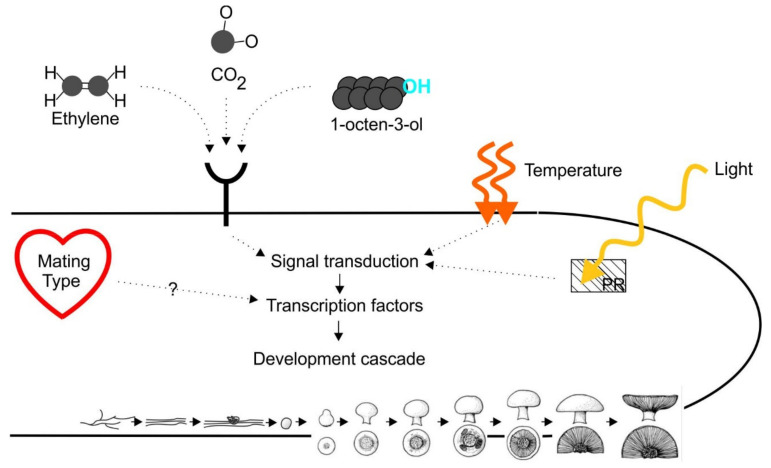
Overview of the main elements in the regulatory system involved in the initiation of the formation of primordia in basidiomycetes. PR = Photoreceptor. (adapted from Nagy et al. [4]). Initiation of fruiting body development in *A. bisporus* is influenced by carbon dioxide, temperature and the removal of a self-produced inhibitor (either ethylene or C_8_-compounds or perhaps both?). Light is not known to be involved in the fruiting process of *A. bisporus*. Details on how these factors link to mating type and transcription factors are presently not known.

According to Nagy et al. [4], the initiation of sexual reproduction in fungi comprises universally conserved mechanisms and they provide an overview of the main parts of the regulatory framework involved on the development of fruiting bodies in fungi (Figure 4). *A. bisporus* mating is determined by an unifactorial mating type system [84], although there are reports of monokaryotic fruiting [85]. Commercial strains of *A. bisporus* are exclusively of the *bisporus* variety which produces spores containing 2 nuclei of opposite mating type and which are immediately capable of forming fruiting bosdies after germinating and establisment of vegetative mycelium, provided that the environmental cues are allowing it [86]. Fruiting is induced by lowering the levels of carbon dioxide and the temperature, relative humidity and speed of air circulation in a growing room. Idnurm & Heitman [87] demonstrated that blue/UV light has a role in controlling fungal development and that this is an ancient process that predates the divergence of the fungi into the ascomycete and basidiomycete phyla. 

The importance of light for fungal development has been reviewed extensively [88,89,90]. Even though the genome of *A. bisporus* contains sequences coding for white collar photoreceptors-like protein (Photo Rexeptor), to our knowledge no research has been performed on the function of these proteins (or other light receptors). In contrast to other basidiomycetes, However, no light is required to induce the fruiting process in *A. bisporus* and the further development of the mushrooms. 

When venting the growing room, the level of carbon dioxide is lowered. Next to this it is assumed that levels of ethylene and 8-carbon compounds are lowered, either by diffusing away from the mycelium or by consumption of micro-organisms. Both these compounds have been postulated to inhibit the formation of primordia in *A. bisporus*. According to Eastwood et al. [20], 1-octen-3-ol and temperature reduction are the master control switches for the complete morphogenesis from vegetative mycelium to fruiting body formation. The 1-octen-3-ol is produced by the mycelium and according to Noble et al. [45] the bacteria in the casing soil use 1-octen-3-ol as a nutrient source (thus acting as a sink for the 1-octen-3-ol that is produced by the mycelium). According to Pfeil and Mumma [53] the production of 1-octen-3-ol during a crop cycle is very low. Furthermore, from the studies of Noble et al. [45] it is clear that it was very difficult to discriminate the production of 1-octen-3-ol amongst all other volatile organic compounds that were present in their microcosm studies. Ethylene is the other volatile organic compound that is postulated to influence the development of primordia. From their micro-array study, Eastwood et al. [20] were not able to pick up a signal for a role of ethylene. Nevertheless, Zhang et al. [57] demonstrated that lowering the production of ethylene and its precursor 1-aminocyclopropane-1-carboxylic acid (ACC) generated twice as much primordia which appeared 3–5 days sooner than the *A. bisporus* wild type strain. This contrasts with the results of Kurtzman [56] who found that inhibiting the production of ethylene by adding 0.8 mM cobalt to either the compost or the casing soil, delayed the production of fruiting bodies by a number of days as compared to the control without cobalt added. Clearly more research is needed to provide clarity on this matter. We assume that ethylene effects the mycelium in the casing soil that is involved in formation of primordia. Turner et al. [47] demonstrated that there is about 8–10 times higher production of ethylene from the compost in comparison to the casing soil. Chen et al. [35] demonstrated that the casing soil contains bacteria that are able to degrade a precursor for the production of ethylene. This raises the question whether the bacteria in the casing soil are able to lower the ethylene production in the casing soil by a factor 8–10 or whether the mycelium produces less mycelium in the casing soil (regardless the presence of bacteria). So even though a role for ethylene has convincingly been demonstrated in the development of primordia by *A. bisporus*, still a lot of questions remain on the exact moment in the morphogenetic process at which it exerts its effect. For 1-octen-3-ol a role is postulated in the transition from cords to hyphal knots (Figure 3). Is ethylene active at the same position in the morphogenetic process? In summary, there is still a lot unknown on the exact way the environmental cues (carbon dioxide, temperature, 1-octen-3-ol, ethylene) exert their effect. The receptors are used by *A. bisporus* to sense the presence of 1-octen-3-ol or ethylene still need to be identified. Nagy et al. [4] mention that in general nutrient availability (one of the internal cues in Figure 4) is an important signal for sex in fungi and that nutrient-sensing pathways regulate sexual development through the mating-type genes. However, in case of *A. bisporus* there are no indications of nutrient limitation, albeit that the casing soil on which the mushrooms develop is low in nutrients. However, the mycelium in the casing soil forms a network with the mycelium in the compost (which is nutrient rich). However, nutritional competition might play a role in regulating the number of primordia that eventually develop into mature mushrooms [17]. Nevertheless, we still do not fully understand the mechanism that regulates the outgrow of only a selection of the abundant amount of primordia into mature mushrooms.

Linking of signals from receptors to transcription factors is likely to be mediated by G protein-coupled receptors [91], although no studies have ever been performed with *A. bisporus*. Ohm et al. [71] proposed a model for fructification in *S. commune*, based on a number of transcription factors believed to be common to basidiomycetes. Pelkmans et al. [79] studied the expression of these transcription factors in *A. bisporus* and found similarities to the expression profile in *S. commune*. However, overexpression of transcription factor *c2h2* in *A. bisporus* resulted in cultivation experiments only in a minor effect on fructification (a somewhat earlier production peak). This would suggest that although there are similarities in the regulatory gene circuits underlying the initiation of fruiting body development, there are species specific variations in this program.

## 7. Conclusions

When comparing the various studies on the environmental influences on the formation of primordia by *A. bisporus*, big differences become evident in the level of detail at which the various authors describe the morphological development. Eastwood et al. [20] are very accurate in their descriptions of the developmental stage at which they sampled their fungal material for analysis of the transcribed genes. On the other hand, Chen et al. [35] do not provide a lot of details. They report on the presence of primordia, that are either formed in large numbers or formed in low numbers, without defining what they consider to be a primordium. This makes it rather difficult to make a good comparison between studies. Apparently, the influence of the self-inhibitor is taking its effect during the very early (and poorly visible) stages of the process; formation of cords and hyphal knots (see Figure 1). Yet these very early stages are not often included in studies. We would like to advocate that in future studies, the early stages of the fruiting process are included, using a similar nomenclature as described by Eastwood et al. [20] and shown in Figure 1. It would provide more detail on the exact developmental stage that was sampled. Next to this, we think that studies into the transcription factors involved in the fruiting process, their interrelationship with temperature, carbon dioxide levels and the self-inhibiting factor, along with their relationship with the mating type gene, should be highly encouraged. In conclusion, the contours of a full model explaining the influence of environmental and chemical factors on fruiting in *A. bisporus* are emerging from literature, although there are still significant blind spots
